# Somatic growth outcomes in response to an individualized neonatal sodium supplementation protocol

**DOI:** 10.21203/rs.3.rs-3911085/v1

**Published:** 2024-02-05

**Authors:** Lyndsay Harshman, Elliot Stalter, Silvia Verhofste, John Dagle, Emily Steinbach, Patrick Ten Eyck, Linder Wendt, Jeffrey Segar

**Affiliations:** University of Iowa Stead Family Department of Pediatrics; University of Iowa

## Abstract

**Objective:**

Evaluate the impact of a sodium (Na) supplementation protocol based upon urine Na concentration on growth parameters and morbidities.

**Study Design:**

Retrospective cohort study of infants 26^0/7^-33^6/7^ weeks gestational age (GA) cared for before (2012–15, n = 225) and after (2016–20, n = 157) implementation of the protocol. Within- and between-group changes over time were assessed using repeated measures generalized linear models.

**Results:**

For infants 26^0/7^-29^6/7^ weeks GA, utilization of the protocol was associated with increased mean body weight z-score at 8-weeks postnatal age, increased mean head circumference z-score at 16-weeks postnatal age, and decreased time on mechanical ventilation (all p < 0.02). No impact on growth was identified for infants 30–33^6/7^ weeks GA. Incidences of hypertension, hypernatremia, bronchopulmonary dysplasia, and culture positive sepsis were unaffected by the protocol.

**Conclusion:**

Protocolized Na supplementation results in improved growth and reduced time on invasive mechanical ventilation in extremely preterm infants without increasing incidence of morbidities.

## Introduction

Sodium (Na) is the major cation of the extracellular fluid, and its homeostasis is primarily regulated by the kidney. Preterm infants are at high risk of chronic negative Na balance due to developmentally immature nephrons, which lack adequate mechanisms for Na reabsorption in the proximal and distal convoluted tubules [1–3]. Chronic total body Na depletion deleteriously impacts somatic growth in infants and children [4–7], and poor growth during the first months of life may impair short- and long-term neurodevelopment [8–10].

The American Academy of Pediatrics currently recommends a Na intake of 3–5 mEq/kg/day for clinically stable preterm infants with birth weight < 1500 grams [11]. However, recent studies suggest that many preterm infants require significantly more dietary Na to account for large urinary Na losses [12–15]. Recent recommendations from the European Society of Paediatric Gastroenterology Hepatology and Nutrition’s Committee on Nutrition acknowledge this increased requirement, suggesting a range of 3–8 mEq/kg/d in select populations [16].

Serum Na concentration is commonly used to prompt initiation of neonatal Na supplementation, but *in vivo* data suggest serum Na concentration to be a relatively poor predictor of total body Na status [17–19]. Segar et al developed an algorithm for individualized enteral Na supplementation of preterm infants born at various gestational ages based upon urine Na concentrations beginning at 2 weeks postnatal age [15]. Clinical application of a protocol based upon this algorithm was initiated in the University of Iowa Stead Family Children’s Hospital Neonatal Intensive Care Unit (UI SFCH NICU) in January 2016. The first 40 infants born between 26^0/7^-29^6/7^ weeks GA and cared for according to the protocol demonstrated increased mean total body weight z-score at 8 weeks postnatal age compared to recent historical controls (n = 50) without significant differences in caloric or protein intakes [13]. Based on these limited, preliminary results, the protocol has continued as standard of care for all infants born between 26^0/7^-33^6/7^ weeks GA.

The objectives of the present study were to 1) utilize more robust and longitudinal cohorts to evaluate somatic growth of preterm infants receiving early-life Na supplementation according to the protocol and 2) identify potential morbidities associated with use of the protocol.

## Methods

### Na supplementation protocol

The protocol has been previously described by Segar et al and is displayed in Table 1 [13]. In brief, receipt of supplementation required the presence of a spot urine Na concentration less than that anticipated for gestational and postnatal age or a serum Na value ≤ 132 mEq/L beginning at two weeks postnatal age. Infants found to have urine or serum Na concentrations below these threshold values initially received 4 mEq/kg/day of enteral Na supplementation above current dietary Na intake with weekly adjustment to account for weight gain. Na supplementation could be provided as NaCl or Na citrate at the discretion of the provider. Urine Na was reassessed every two weeks and Na supplementation was increased by an additional 2 mEq/kg/day if the urine Na concentration was below the anticipated value for gestational and postnatal age.

### Study Population and Design

A retrospective review of patient data was approved with waived consent by the University of Iowa Institutional Review Board (202203591). The study sample included infants treated in the UI SFCH NICU and subsequently evaluated in the University of Iowa High-Risk Infant Follow-up Clinic. Electronic medical record (EMR) data were obtained for 461 infants born between 26^0/7^-29^6/7^ weeks GA and 357 infants born between 30^0/7^-33^6/7^ weeks GA admitted to the UI SFCH NICU between 2012–2020 and 2014–2017, respectively. The narrower time window for infants 30^0/7^-33^6/7^ weeks GA was purposeful to limit the cohort size to that similar for the between 26^0/7^-29^6/7^ weeks GA population. Infants describe in the original publication describing the urine Na algorithm (26^0/7^ – 29^6/7^ weeks gestation; n = 50 pre-algorithm, n = 40 post-algorithm) are included in this larger cohort study [13].

Infants diagnosed with conditions associated with delayed growth or which contraindicated Na supplementation were excluded. Specifically, infants with underlying cardiovascular disease (tetralogy of Fallot, atrial septal defect, ventricular septal defect, ventricular dysfunction/hypertrophy, heart failure, pulmonary hypertension, pulmonary artery atresia/stenosis/aneurysm, or pulmonic valve stenosis/regurgitation/dysplasia), kidney disease (chronic kidney disease, bilateral hypoplastic/dysplastic kidneys, bilateral vesicoureteral reflux grade 4 or 5, bilateral hydronephrosis, posterior urethral valves, or infants requiring neonatal dialysis), or intestinal ostomies were excluded from analysis. Additionally, a small number of infants missing vital data or upon whom the protocol was incompletely or incorrectly applied were similarly excluded. After application of exclusion criteria, the 26^0/7^-29^6/7^ weeks GA infants were stratified into 157 pre-protocol (2012–2015) and 225 post-protocol (2016–2020) infants. The 30^0/7^-33^6/7^ weeks GA cohort was similarly divided into 153 pre- and 157 post-protocol infants (Figure).

### Study Procedures

#### Somatic growth, ventilatory support, and length of stay outcomes

To evaluate somatic growth, body weight (g), body length (cm), and head circumference (cm) were abstracted from the EMR and z-scores were calculated using the Fenton Preterm Growth Chart for boys or girls [20, 21]. Growth variables were recorded at 2-, 4-, 6-, 8-, and 16-weeks postnatal age. Each recorded measurement represented the temporally nearest measurement to its specified timepoint and occurred within 1 week of its corresponding time point except the 16-week timepoint, for which measurements within 4 weeks were accepted due to variation in outpatient appointment scheduling. A single measurement was never recorded for two separate timepoints. If two measurements were temporally equidistant from one of the specified time points, a random number generator determined which measurement was recorded. If a single measurement was temporally equidistant from two specified time points, a random number generator similarly determined to which time point it was applied.

Length of stay was defined as the number of days elapsed between an infant’s birth and initial date of discharge from the UI SFCH NICU. Time on invasive mechanical ventilation was defined as the total number of days during which ventilation requiring an orally inserted endotracheal tube was utilized prior to discharge. Discontinuous periods of invasive mechanical ventilation were summed to determine an infant’s total time on invasive mechanical ventilation when applicable.

#### Additional Morbidities

Diagnoses of systemic hypertension, bronchopulmonary dysplasia, and culture positive sepsis were recorded for an infant if present on the EMR discharge problem list. Hypertension was also recorded if use of antihypertensive medication was documented within the EMR. Diagnosis of bronchopulmonary dysplasia was defined by the prevailing diagnostic criteria accepted by the NICHD Neonatal Research Network during the study period [22]. Hypernatremia (serum Na value ≥ 150 mEq/L) was identified by review of laboratory data within the EMR.

### Statistical analyses

Baseline demographics of infants from pre- and post-protocol cohorts were reported as medians (interquartile ranges) and compared using the Wilcoxon rank sum test. The repeated measures generalized linear modeling framework was used to evaluate the impact of the protocol on somatic growth at 8- and 16-weeks postnatal age stratified by GA level. Models were fit using patient data from 0-, 2-, 4-, 6-, 8-, and 16-weeks postnatal age. The primary predictors of focus were week (categorical), cohort, and their interaction, but GA, sex, and birthweight were also included as control measures. Point and interval estimates are reported for within- and between-cohort mean differences for bodyweight z-score from weeks 2–8 and head circumference z-score from weeks 2–16 along with p-values. The generalized linear modeling framework was used to assess between-cohort differences in time on invasive mechanical ventilation and length of stay stratified by GA level. The primary predictor of focus was cohort, but GA, sex, and birthweight were also included as control measures. Point and interval estimates are reported for between-group mean ratios (mechanical ventilation) or differences (length of stay) along with p-values.

The logistic regression framework was used to investigate whether adverse health outcomes were associated with protocol application. For each GA group, models were fit using cohort, GA, sex, and birthweight to predict systemic hypertension, hypernatremia, bronchopulmonary dysplasia, and culture positive sepsis. Within-cohort rates are reported along with the adjusted p-value for between-cohort significance. Statistical analysis was performed using SAS, The R Project for Statistical Computing, and RStudio. For all tests, a p-value of < 0.05 was concluded to be statistically significant.

## Results

### Demographics

Baseline demographics of study cohorts are outlined in Table 2. No significant demographic differences were identified between infant cohorts within either GA group.

### Somatic growth, ventilatory support, and length of stay

A summary of somatic growth, length of stay, and ventilatory support outcomes for all cohorts is provided in Table 3. Within the 26^0/7^-29^6/7^ weeks GA cohort, post-protocol infants experienced a significantly increased positive change in z-score between 2- and 8-weeks postnatal age (p = 0.002, 95% CI = 0.06, 0.25) and an increased positive change in mean head circumference z-score between 2- and 16-weeks postnatal age (p = 0.013, 95% CI = 0.07, 0.58). Post-protocol infants also spent significantly fewer days on invasive mechanical ventilation than pre- infants (p = 0.013, 95% CI = 0.50, 0.92). No significant differences in change in body length z-score or length of stay were identified between pre- and post-protocol infants.

Within the 30^0/7^-33^6/7^ weeks GA cohort, no differences between pre- and post-protocol infants were identified with respect to weight gain, body length, head circumference, or time on invasive mechanical ventilation. Post infants experienced a greater length of stay (p < 0.001, 95% CI = 3.1, 10.0).

### Additional morbidities

A summary of adverse health outcomes for all cohorts is provided in Table 4. Use of the protocol was not associated with increased incidence of systemic hypertension, hypernatremia ≥ 150 mEq/L, bronchopulmonary dysplasia, or culture positive sepsis in either GA cohort.

## Discussion

Contemporary advances in neonatal nutrition practices have improved preterm infant growth velocity but nearly half of very low birth weight infants continue to experience impaired postnatal growth [23]. Isemann et al and Segar et al have independently demonstrated improved preterm infant growth following enteral Na supplementation above that typically provided in a clinical setting, suggesting body Na depletion may contribute to postnatal growth failure [12, 13]. The present study sought to expand upon the findings of Segar et al by utilizing a significantly larger, more longitudinal sample of infants and examining a larger number of variables to more robustly evaluate the efficacy and safety of the protocol. Our findings demonstrate application of the protocol in 26^0/7^-29^6/7^ weeks GA infants is associated with improved somatic growth and decreased need for invasive mechanical ventilation without increasing incidences of common morbidities.

Among 26^0/7^-29^6/7^ weeks GA infants, adherence to the protocol was associated with improved somatic growth reflected by change in body weight between 2- and 8-weeks postnatal age and change in head circumference between 2- and 16-weeks postnatal age. While the mechanism by which Na depletion impairs infant growth is not known, Ziegler et al recently demonstrated in an animal model that insufficient early life Na intake results in suboptimal growth and energy utilization due to increased resting energy expenditure [24]. Although increased total body water resulting from increased Na intake cannot be ruled out as a contributor to the increased weight gain observed among post-protocol infants, studies in young animals and human adults indicate 10-fold or greater differences in Na intake have no effect upon total body water expressed as a percent of total body weight [17, 25]. Postnatal growth failure is of significant concern as it has previously been associated with impaired neurodevelopmental outcomes [8–10]. Although the present study did not assess neurodevelopment, the increased head circumference growth observed among the 26^0/7^-29^6/7^ weeks GA post-protocol infants is intriguing since poor cephalic growth in the neonatal period has been associated with motor and cognitive delays at 16 to 36 months of life [26].

Notably, application of the protocol had no effect on growth in infants born at ≥ 30 weeks GA. This finding is not surprising given that even the most premature infants within this cohort were at approximately 33 weeks postmenstrual age upon initiation of Na supplementation, at which point growth failure secondary to Na depletion is far less likely due to more mature renal tubular Na reabsorption mechanisms and resultant decreased urine Na losses [2, 27].

Application of the protocol was associated with decreased time on invasive mechanical ventilation among infants 26^0/7^-29^6/7^ weeks GA. While we cannot directly attribute this finding to increased Na intake or improved weight gain, the activity of Na channels/transporters, which are likely altered with Na depletion, are important for cell proliferation and maintenance of lung function [28–30]. The role of Na homeostasis in regulating postnatal lung development requires further investigation. Alternatively, decreased time on mechanical ventilatory support may be related to changes in clinical respiratory management instituted within the study period [31].

Implementation of the protocol was not associated with increased incidence of bronchopulmonary dysplasia, culture positive sepsis, systemic hypertension, or hypernatremia ≥ 150 mEq/L, suggesting application of the protocol does not result in excessive Na administration or its potential sequelae. Of note, incidence of culture positive sepsis among 26^0/7^-29^6/7^ weeks GA infants was approximately 50% lower after initiation of the protocol. Although this difference was not significant, it is consistent with findings of Isemann et al, who demonstrated Na supplementation of 4 mEq/kg/d during day of life 7–35 significantly decreased incidence of late onset sepsis and necrotizing enterocolitis [12]. There are emerging data regarding the impact of Na in the regulation of immune function and the inflammatory response, but whether total body Na status impacts preterm infant infection risk requires further investigation [32, 33].

We acknowledge several limitations of the present study. Clinical application of the protocol within the UI SFCH NICU occurred by agreement of attending neonatologists after review of assembled pilot data, not the findings of a clinical trial. The protocol’s urine Na concentration threshold values are intentionally conservative and may not represent optimal concentrations for identifying Na deficiency in preterm infants. The protocol also relies on single spot urine Na concentrations, which may not reflect daily urine Na losses. Additionally, nutritional intake was also not evaluated, although no differences in caloric or protein intake were observed between the pre- and post-protocol cohorts of the original publication describing the protocol [13]. Changes in clinical practice over the course of the study period may have additionally impacted outcomes. For example, the increased length of stay observed in the very/moderately preterm post infants is likely related to changes in screening and management protocols concerning pulmonary hypertension, atrial septal defects, feeding issues, and retinopathy of prematurity instituted over the course of the study period. Inability to ascertain discharge home versus transfer to another NICU, which occurs for only a small number of patients, may also impact length of stay data. Lastly, there is a risk for selection bias as infants who died prior to hospital discharge were not included in the study.

The findings of the present study strengthen and extend previous work regarding use of a physiologically based Na supplementation protocol in preterm infants in the clinical setting. This approach to Na supplementation appears to be a safe, individualized method to optimize early life postnatal growth. We await the results of a randomized trial examining the impact of the protocol on growth, total body water, and energy expenditure before recommending wider adoption of the practice (ClinicalTrials.gov NCT 03889197). Future studies are also required to comprehensively evaluate the impact of targeted neonatal Na supplementation on long term cardiometabolic and neurodevelopmental outcomes.

## Figures and Tables

**Figures 1:**
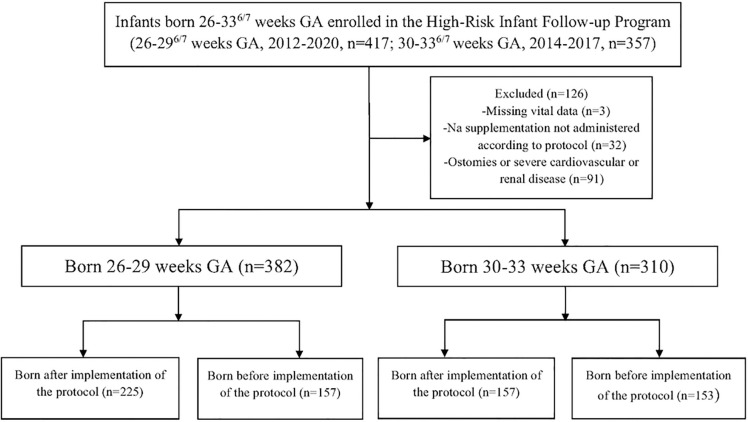
Flow diagram demonstrating subject enrollment, application of exclusion criteria, and cohort formation.

**Table 1:** Enteral Na supplementation protocol, adapted from Segar et al [13].

	2 wk PA	4 wk PA	6 wk PA	8 wk PA	10 wk PA	12 wk PA
**22–25 wk GA**	-	≤ 50 mEq/L	≤ 40 mEq/L	≤ 40 mEq/L	≤ 40 mEq/L	≤ 30 mEq/L
**26–29 wk GA**	≤ 40 mEq/L	≤ 40 mEq/L	≤ 40 mEq/L	≤ 30 mEq/L	-	-
**30–34 wk GA**	≤ 40 mEq/L	≤ 30 mEq/L	-	-	-	-

Values represent urine [Na], obtained every other week beginning at approximately day 14 postnatal age (PA). If urine [Na] is below the value, Na supplementation is initiated at 4 mEq/kg/day above current Na intake. Na supplementation is increased by 2 mEq/kg at subsequent time points if urine Na is less than the urine [Na] goal. Urine Na measurements are not obtained within 48 hours of use of a diuretic agent. Na supplementation or determination of urine [Na] is considered if serum [Na] is ≤ 132 mEq/L prior to the first scheduled urine [Na] determination. Na supplementation is provided if serum [Na] is ≤ 132 mEq/L, regardless of urine [Na], unless there is evidence of acute fluid overload. Na supplementation is continued unless serum [Na] is > 144 mEq/L. Protocol driven Na supplementation is discontinued at 38 weeks’ postmenstrual age.

**Table 2:** Summary table of baseline demographics of study cohorts.

		Institution of the protocol		
Mean	GA	Pre	Post	p-value
**GA (wk, median (IQR))**	26–29 weeks	28 (27, 29)	28 (27, 29)	0.3
	30–33 weeks	32 (31, 33)	32 (31, 33)	0.4
**Sex (N male, N female)**	26–29 weeks	82, 75	116, 109	0.9
	30–33 weeks	92, 61	92, 65	0.8
**Birthweight (g, median (IQR))**	26–29 weeks	1070 (912,1244)	1125 (936,1315)	0.08
	30–33 weeks	1715 (1497, 1950)	1683 (1457, 1962)	0.5

GA, gestational age

**Table 3:** Somatic growth, ventilatory support, and length of stay outcomes.

		Institution of the protocol				
Mean	GA	Pre	Post	Mean Difference	95% CI	p-value
**Change in body weight z-score, 2–8 weeks**	26–29 weeks	−0.07	0.08	0.15	[0.06, 0.25]	**0.002**
	30–33 weeks	0.42	0.5	0.07	[−0.16, 0.30]	0.5
**Change in head circumference z-score, 2–16 weeks**	26–29 weeks	−0.39	−0.07	0.32	[0.07, 0.58]	**0.013**
	30–33 weeks	0.12	−0.43	−0.55	[−1.09, 0.00]	0.051
**Time on invasive mechanical ventilation** (d, mean ± SD)	26–29 weeks	14.1 ± 19.0	9.6 ± 13.8	0.68*	[0.50, 0.92]	**0.013**
	30–33 weeks	0.6 ± 1.2	0.7 ± 1.2	1.09*	[0.71, 1.66]	0.7
**Length of stay** (d, mean ± SD)	26–29 weeks	73.6 ± 25.5	77.9 ± 23.4	4.3	[−0.7, 9.2]	0.091
	30–33 weeks	34.6 ± 15.5	41.2 ± 15.4	6.6	[3.1, 10.0]	**< 0.001**

* Mean Ratio

**Table 4:** Incidence of adverse event outcomes.

		Institution of the protocol		
Measure	GA	Pre	Post	p-value
**Systemic Hypertension**	26–29 weeks	2.6%	2.7%	0.8
	30–33 weeks	0%	0%	-
**Hypernatremia ≥ 150 mEq/L**	26–29 weeks	21.0%	15.1%	0.2
	30–33 weeks	2.6%	3.8%	0.7
**Bronchopulmonary Dysplasia**	26–29 weeks	40.1%	42.7%	0.7
	30–33 week	5.2%	4.5%	0.8
**Culture Positive Sepsis**	26–29 weeks	14.6%	9.8%	0.15
	30–33 week	1.3%	2.5%	0.7
